# Auxin promotes hypocotyl elongation by enhancing BZR1 nuclear accumulation in *Arabidopsis*

**DOI:** 10.1126/sciadv.ade2493

**Published:** 2023-01-04

**Authors:** Zipeng Yu, Jinxin Ma, Mengyue Zhang, Xiaoxuan Li, Yi Sun, Mengxin Zhang, Zhaojun Ding

**Affiliations:** The Key Laboratory of Plant Development and Environmental Adaptation Biology, Ministry of Education, School of Life Sciences, Shandong University, Qingdao, Shandong, China.

## Abstract

Auxin and brassinosteroids (BRs) are two major growth-promoting phytohormones that shape hypocotyl elongation; however, the cross-talk between auxin and BR in this process is not fully understood. In this study, we found that auxin-induced hypocotyl elongation is dependent on brassinazole-resistant 1 (BZR1), a core BR signaling component. Auxin promotes BZR1 nuclear accumulation in hypocotyl cells, a process dependent on mitogen-activated protein kinase 3 (MPK3) and MPK6, which are both activated by auxin and whose encoding genes are highly expressed in hypocotyls. We determined that MPK3/MPK6 phosphorylate and reduce the protein stability of general regulatory factor 4 (GRF4), a member of the 14-3-3 family of proteins that retain BZR1 in the cytoplasm. In summary, this study reveals the molecular mechanism by which auxin promotes hypocotyl elongation by enhancing BZR1 nuclear accumulation via MPK3/MPK6-regulated GRF4 protein stability.

## INTRODUCTION

Hypocotyl elongation is a prerequisite for freshly germinated seedlings to reach the soil surface and emerge into sunlight. The initial darkness experienced by the seedlings in the soil is an important signal that promotes a developmental program known as skotomorphogenesis (*1*, *2*). In the dark, transcription factors from the phytochrome-interacting factor (PIF) family induce the expression of numerous cell elongation–related genes, and the *pifQ* (*pif1*,* pif3*,* pif4*, and* pif5*) quadruple mutant exhibits a constitutive photomorphogenic phenotype characterized by the short hypocotyl of etiolated (dark-grown) seedlings (*3*), indicating the central role of PIFs in hypocotyl elongation. In addition to skotomorphogenesis-activated PIFs, the expression of cell elongation–related genes is also closely related to auxin-activated auxin response factor 6 (ARF6) and brassinosteroid (BR)–activated brassinazole-resistant 1 (BZR1) (*4*, *5*), highlighting the essential roles of auxin and BR in hypocotyl elongation.

The role of auxin in hypocotyl elongation was established over a century ago and has since been formalized as the auxin-induced “acid growth theory” (*6*, *7*). Genetic approaches have greatly improved our understanding of auxin signaling, with many auxin signaling mutants exhibiting defects in hypocotyl elongation (*8*–*12*), confirming the important role of auxin in hypocotyl elongation. Isolated plant stem segments can be induced to elongate by auxin within minutes, illustrating the speed of the underlying response (*13*). Auxin promotes apoplastic acidification by regulating plasma membrane (PM)–localized H^+^-dependent adenosine triphosphatases (AHAs), which activates the cell wall remodeling-related expansins (*14*), xyloglucan endotransglucosylase/hydrolases (*15*), and pectin methylesterases (*16*), eventually causing cell expansion and hypocotyl/stem elongation. Auxin not only induces *AHA* transcription (*17*, *18*) but also attenuates the endocytosis of their encoded proteins and accelerates AHA exocytosis (*19*), ensuring adequate density of AHAs at the PM. In addition, D–clade type 2C protein phosphatase (PP2C.D) is a phosphatase that dephosphorylates and inhibits AHAs activity, but its activity can be inhibited by auxin-induced small auxin up RNAs (*20*, *21*). The higher transcription of *AHA*s or the increased activity of AHAs, which depends on the canonical auxin signaling pathway mediated by transport inhibitor response 1/auxin signaling F-box (*6*), eventually increases apoplastic acidification and promotes cell expansion. Auxin was also recently shown to phosphorylate AHAs in a transmembrane kinase (TMK1/TMK4)–dependent manner (*22*) to enhance apoplastic acidification and cell elongation in the hypocotyl.

In addition to auxin, BRs also play a well-established role in hypocotyl elongation (*23*–*25*). BZR1, a key transcription factor in BR signaling, plays an irreplaceable role in BR-induced hypocotyl elongation. In the absence of BR, BZR1 is phosphorylated by BR-insensitive 2 (BIN2) (*25*), before being transported from the nucleus to the cytoplasm by 14-3-3 proteins by an unknown mechanism (*26*, *27*) to switch off BZR1-mediated BR signaling. BR treatment recruits cytosolic BZR1 to the nucleus and, together with PIF4, induces the expression of cell elongation–related genes (*5*). Expression of the dominant mutant of BZR1 (*bzr1-1D* or *BZR1^S173A^*) in BR-deficient mutants suppresses the short hypocotyl phenotype of these mutants (*27*–*29*), suggesting the essential role of BZR1 in BR-induced hypocotyl elongation.

Accumulating evidence supports a synergistic role for auxin and BRs in hypocotyl elongation (*4*, *30*); ARF6 even shares almost half of its target genes with BZR1 (*4*). In addition, the PIF4-ARF6-BZR1 regulatory module functions interdependently to control the expression of cell elongation–related genes in the hypocotyl (*4*). However, another study reported that auxin inhibits the nuclear accumulation of BZR1 in the root stem cell niche (*31*), suggesting antagonistic roles for auxin and BRs in root stem cell niche identity. Therefore, the detailed molecular mechanism behind the interactions between auxin and BR signaling remains to be further investigated.

In this study, we investigated the relationship between auxin-induced hypocotyl elongation and BZR1-mediated BR signaling and found that BZR1 dysfunction blocks auxin-induced hypocotyl elongation. In contrast to auxin-inhibited BZR1 nuclear accumulation in the root stem cell niche, we observed that auxin enhances the nuclear accumulation of BZR1 in hypocotyl cells. Immunoprecipitation followed by mass spectrometry (IP-MS) using seedlings overexpressing *BZR1-YFP* identified mitogen-activated protein kinase 3 (MPK3), which can be activated by auxin, as a candidate BZR1 interacting partner. Although both MPK3 and MPK6 contributed to auxin-induced hypocotyl elongation, they did not directly interact with BZR1. We determined that general regulatory factor 4 (GRF4), a member of the 14-3-3 family, acts as a bridge protein between MPK3/MPK6 and BZR1. Phosphorylation of GRF4 by MPK3 and MPK6 at serine-248 (S248) promoted GRF4 degradation by the 26*S* proteasome, thus leading to the nuclear localization of BZR1 and inducing hypocotyl elongation.

## RESULTS

### Auxin-induced hypocotyl elongation depends on BZR1

**Fig. 1.**
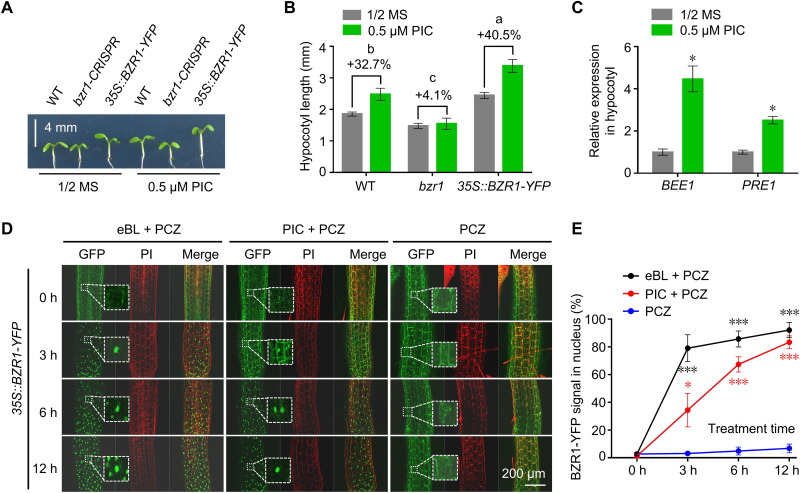
Auxin promotes hypocotyl elongation dependent on BZR1. (**A**) Hypocotyl phenotypes of the wild-type (WT, Columbia-0), *bzr1*, and *35S::BZR1-YFP* seedlings grown on half-strength Murashige and Skoog (MS) medium (1/2 MS) with or without 0.5 μM picloram (PIC) for 6 days. Scale bar, 4 mm. (**B**) Mean hypocotyl length of the seedlings shown in (A). The percentages indicate the promoting effect of auxin (+ PIC) on hypocotyl elongation. *P* < 0.05 by one-way analysis of variance (ANOVA). (**C**) Relative *BEE1* and *PRE1* transcript levels in WT hypocotyls grown on half-strength MS medium with or without 0.5 μM PIC for 6 days. *P < 0.05 by Student’s *t* test. (**D**) Subcellular localization of BZR1–yellow fluorescent protein (YFP) in hypocotyl cells of *35S::BZR1-YFP* seedlings grown on half-strength MS medium with 0.2 μM propiconazole (PCZ) for 6 days and then treated with 0.2 μM PCZ, 0.2 μM PCZ + 1 μM epi-brassinolide (eBL), or 0.2 μM PCZ + 1 μM PIC for 3, 6, and 12 hours, respectively. Dotted lines indicate enlarged images. Scale bar, 200 μm. PI, propidium iodide. (**E**) Percentage of BZR1-YFP signal in the nucleus based on quantification of the signal in (D) by ImageJ software. *P < 0.05 and ***P < 0.001 by Student’s *t* test. h, hours.

Exogenous auxin treatment can promote hypocotyl elongation (*22*, *32*, *33*). To clarify whether the promoting effect of auxin depends on BZR1, a core BR signaling component, we examined the hypocotyl phenotype of *bzr1* and *35S::BZR1-YFP* seedlings (*34*) when treated with the synthetic auxin analog picloram (PIC). While hypocotyls elongated upon PIC treatment in wild-type (WT) seedlings, this effect was almost abolished in *bzr1* and enhanced in *35S::BZR1-YFP* seedlings (Fig. 1, A and B), suggesting that auxin-promoted hypocotyl elongation depends on BZR1. Consistent with this, PIC treatment raised the transcript levels of *BR-enhanced expression* *1* (*BEE1*) and *paclobutrazol resistance 1* (*PRE1*), two BZR1 downstream genes associated with hypocotyl cell elongation (Fig. 1C). However, PIC treatment did not affect the transcript levels of *BZR1* (fig. S1A) and 1-naphthaleneacetic acid (NAA) treatment only slightly enhanced BZR1 protein stability (fig. S1B), which might not be sufficient to explain the critical role of BZR1 in auxin-induced hypocotyl elongation (Fig. 1, A and B). These observations prompted us to evaluate BZR1 subcellular localization with and without PIC treatment in hypocotyl cells. Propiconazole (PCZ) is an effective inhibitor of BR biosynthesis (*35*) that leads to an enrichment of BZR1 in the cytoplasm, making any effect of BR or auxin on BZR1–yellow fluorescent protein (YFP) subcellular localization very clear. Consistent with previous observations (*26*), the synthetic BR analog epi-brassinolide (eBL) treatment rapidly induced the relocalization of cytosolic BZR1-YFP to the nucleus. Notably, PIC also promoted a strong BZR1-YFP accumulation in the nucleus, similar to that seen upon eBL treatment after 12 hours of treatment (Fig. 1, D and E). In summary, auxin-induced hypocotyl elongation depends on BZR1, and auxin-induced BZR1 nuclear accumulation might be involved.

### Auxin-induced BZR1 nuclear accumulation and hypocotyl elongation depends on MPKs

**Fig. 2.**
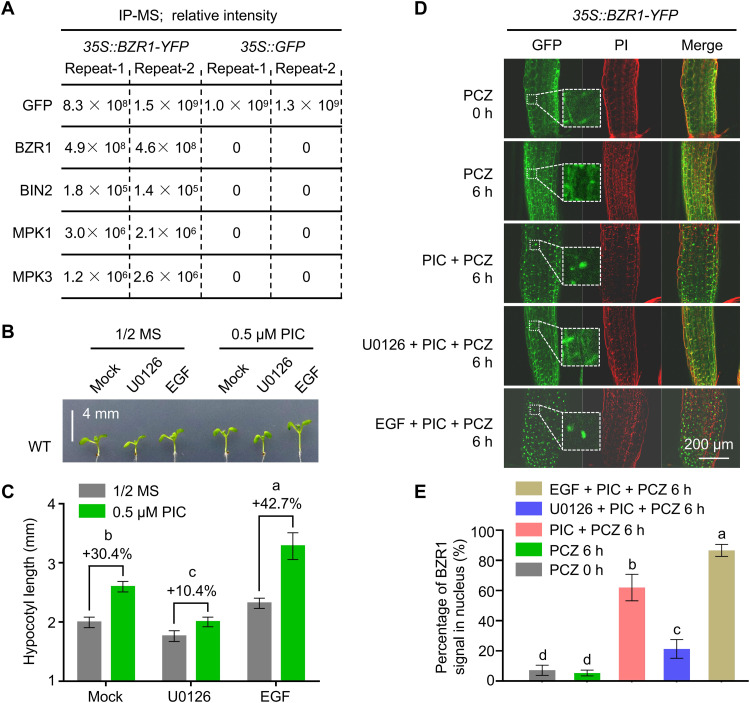
MPKs promote the nuclear accumulation of BZR1. (**A**) Several BZR1-interacting proteins, identified by IP-MS (two technical replicates) using 6-day-old *35S::BZR1-YFP* and *35S::GFP* seedlings. (**B**) Hypocotyl phenotype of 6-day-old WT seedlings grown on half-strength MS medium with 5 μM 1,4-diamino-2,3-dicyano-1,4-bis[2-aminophenylthio]butadiene (U0126) or epidermal growth factor (EGF; 50 ng/ml) and treated with or without 0.5 μM PIC. Scale bar, 4 mm. (**C**) Mean hypocotyl length of the seedlings shown in (B). The percentages indicate the promoting effect of auxin on hypocotyl elongation. *P* < 0.05 by one-way ANOVA. (**D**) Subcellular localization of BZR1-YFP in hypocotyl cells of *35S::BZR1-YFP* seedlings grown on half-strength MS medium with 0.2 μM PCZ for 6 days and then treated with 0.2 μM PCZ, 0.2 μM PCZ + 1 μM PIC, 10 μM U0126 + 1 μM PIC + 0.2 μM PCZ, or EGF (100 ng/ml) + 1 μM PIC + 0.2 μM PCZ for 6 hours. Dotted lines indicate enlarged images. Scale bar, 200 μm. (**E**) Percentage of BZR1-YFP signal in the nucleus based on quantification of the signal in (D) by ImageJ software. *P* < 0.05 by one-way ANOVA.

We next performed IP-MS with *35S::BZR1-YFP* and *35S::GFP* seedlings to identify proteins that are both associated with auxin signaling and interact with BZR1. Among the candidate BZR1-interacting proteins, we identified the kinase BIN2, which phosphorylates BZR1 (*36*), providing an important control for our experimental conditions (Fig. 2A and table S1). In addition, we detected topless/topless related 1 to 4, which interact with BZR1 and transcriptionally repress BZR1 target genes (*37*), only in the *35S::BZR1-YFP* group but not in the *35S::GFP* control group (table S1). We also identified two protein kinases, MPK1 and MPK3, among the candidate BZR1-interacting proteins. Several members of the MPK family, including MPK1/MPK2/MPK14 (*38*, *39*) and MPK3/MPK6 (*40*), have been shown to be activated by auxin, suggesting that auxin-induced BZR1 nuclear accumulation might be mediated by MPKs.

We thus explored the role of MPKs in auxin-triggered hypocotyl elongation via chemical treatments with 1,4-diamino-2,3-dicyano-1,4-bis[2-aminophenylthio]butadiene (U0126), an MPK inhibitor (*41*), or with epidermal growth factor (EGF), an MPK activator (*42*). We determined that the promoting effect of PIC on hypocotyl elongation diminishes in the presence of U0126 but increases upon cotreatment with EGF and PIC (Fig. 2, B and C), indicating that MPKs contribute to auxin-induced hypocotyl elongation. We also examined BZR1 nuclear accumulation in *35S::BZR1-YFP* seedlings treated with U0126 or EGF together with PIC and PCZ. We observed a decline in auxin-induced BZR1 nuclear accumulation following U0126 treatment, while exogenous EGF application had the opposite effect (Fig. 2, D and E). We concluded that auxin-induced BZR1 nuclear accumulation and hypocotyl elongation require MPKs.

### MPK3/MPK6 play a major role in auxin-induced hypocotyl elongation

**Fig. 3.**
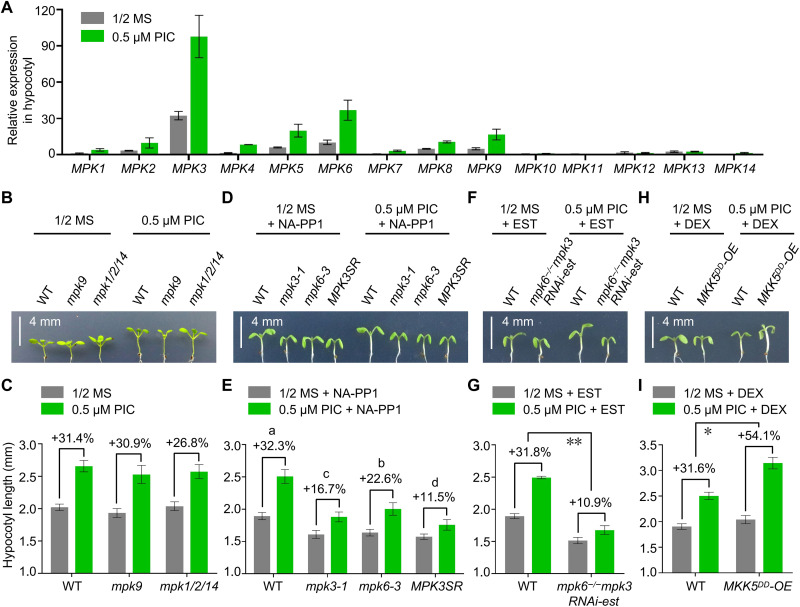
Essential roles of MPKs in auxin-induced hypocotyl elongation. (**A**) Relative *MPK*s transcript levels in the hypocotyls of WT seedlings grown on half-strength MS medium with or without 0.5 μM PIC for 6 days. (**B**, **D**, **F**, and **H**) Hypocotyl phenotypes of WT, *mpk9*, *mpk1 mpk2 mpk14*, *mpk3-1*, *mpk6-3*, *MPK3SR*, *mpk6^−/−^mpk3RNAi-est*, and *MKK5^DD^*-OE seedlings grown on half-strength MS medium or 0.5 μM PIC with or without 0.5 μM 4-amino-1-tert-butyl-3-(1′-naphthyl)pyrazolo[3,4-d]pyrimidine (NA-PP1), 0.02 μM estradiol (EST), or 0.02 μM dexamethasone (DEX) for 6 days. Scale bars, 4 mm. (**C**, **E**, **G**, and **I**) Mean hypocotyl length of seedlings shown in (B), (D), (F), and (H). The percentages indicate the promoting effect of auxin on hypocotyl elongation. For (E), *P* < 0.05 by one-way ANOVA; for (G), **P < 0.01 by Student’s *t* test; and for (I), *P < 0.05 by Student’s *t* test.

MPK1 and MPK3 are two of the candidate BZR1-interacting proteins but belong to different groups: MPK1 belongs to group C MPKs, which also contains MPK2, MPK7, and MPK14, while MPK3 belongs to group A MPKs, with MPK3 and MPK6 as typical representatives (*43*). To identify which MPK subfamily or members play a major role in hypocotyl elongation, we analyzed *MPK* expression levels in hypocotyls. *MPK3* and *MPK6* were the most highly expressed members in hypocotyls, and their transcript levels were further increased by PIC treatment (Fig. 3A), suggesting that group A MPKs may exert a major role in hypocotyl elongation. By contrast, although *MPK1*, *MPK2*, *MPK7*, and *MPK14* transcript levels also increased following PIC treatment, their expression levels remained very low (Fig. 3A), suggesting a weaker contribution of group C MPKs in hypocotyl elongation. To test this hypothesis, we examined the hypocotyl phenotype of various *mpk* mutants with and without PIC treatment. We determined that the *mpk1 mpk2 mpk14* triple mutant (*38*) has a normal hypocotyl length under normal growth conditions and only showed a slight decrease in PIC-induced hypocotyl elongation relative to WT (Fig. 3, B and C), confirming that group C MPKs only exert a modest effect on auxin-induced hypocotyl elongation.

We thus analyzed the role of MPK3 and MPK6 in auxin-induced hypocotyl elongation by examining the hypocotyl phenotype of *mpk3-1* and *mpk6-3* single mutants (*44*–*46*) and *MPK3SR* (*mpk3 mpk6 MPK3pro:MPK3^TG^*), whereby the embryo lethality of the *mpk3 mpk6* double mutant is rescued by a version of MPK3 whose activity is repressed by 4-amino-1-tert-butyl-3-(1′-naphthyl)pyrazolo[3,4-d]pyrimidine (NA-PP1) (*47*). We observed that *mpk3-1* and *mpk6-3* have shorter hypocotyls under normal growth conditions, but this phenotype was exacerbated by PIC treatment; the *MPK3SR* line also showed a more pronounced short hypocotyl phenotype under both conditions, especially upon PIC treatment (Fig. 3, D and E). This result suggested that MPK3 and MPK6 play important and redundant roles in auxin-induced hypocotyl elongation. To confirm these results, we used another conditional *mpk3 mpk6* double mutant line, *mpk6^−/−^mpk3RNAi-est*, in which *MPK3* transcripts are targeted by an RNA interference (RNAi) construct whose expression is induced by estradiol (EST) (*40*). As with *MPK3SR*, *mpk6^−/−^mpk3RNAi-est* also displayed severely attenuated PIC-induced hypocotyl elongation (Fig. 3, F and G). Conversely, the promoting effect of auxin on hypocotyl elongation and BZR1 nuclear accumulation substantially rose in *MKK5^DD^*-OE seedlings (Fig. 3, H and I, and fig. S2), which overexpress a constitutively active MPK kinase 5 (MKK5) to activate MPK3 and MPK6 (*40*, *47*–*50*). In summary, we showed that the MKK5-MPK3/MPK6 signaling module plays a major role in auxin-induced hypocotyl elongation.

### MPK3/MPK6 interact with GRF4, a cytosolic BZR1 retention factor

**Fig. 4.**
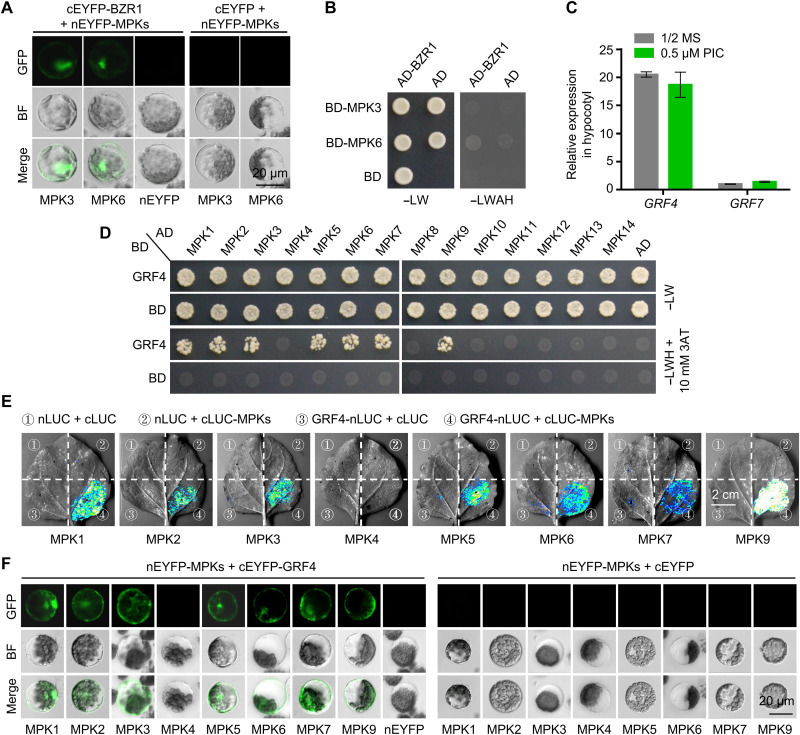
MPK3/MPK6 interact with GRF4. (**A**) Bimolecular fluorescence complementation (BiFC) assay performed in transfected *Arabidopsis* protoplasts showing the interaction between MPK3/MPK6 and BZR1. Scale bar, 20 μm. BF, bright field; cEYFP/nEYFP, c-terminal/n-terminal enhanced yellow fluorescent protein. (**B**) The yeast two-hybrid (Y2H) assay validating the interaction between MPK3/MPK6 and BZR1. AD, activation domain; BD, binding domain. (**C**) Relative *GRF4* and *GRF7* transcript levels in the hypocotyls of WT seedlings grown on half-strength MS medium with or without 0.5 μM PIC for 6 days. (**D**) Y2H assay validating the interaction between MPKs and GRF4. 3AT, 3-amino-1,2,4-triazole. (**E**) Luciferase complementation imaging (LCI) assay performed in *N. benthamiana* leaves monitored the interaction between MPKs and GRF4. Four different combinations, including three negative controls, were separately injected into four different areas of the leaf. Scale bar, 2 cm. nLUC/cLUC, n-terminal/c-terminal luciferase. (**F**) BiFC assay performed in *Arabidopsis* protoplasts validating the interaction between MPKs and GRF4. Scale bar, 20 μm. −LW, without leucine and tryptophan; −LWAH, without leucine, tryptophan, adenine, and histidine.

We tested whether MPK3/MPK6 interact with BZR1. Although we observed an interaction by bimolecular fluorescence complementation (BiFC) and co-IP assays (Figs. 2A and 4A), we saw no evidence of an interaction by yeast two-hybrid (Y2H) assay (Fig. 4B). These results suggested that MPK3/MPK6 and BZR1 might interact indirectly and require a bridging protein, which would help explain the lack of interaction in yeast but the clear interaction detected in planta.

A reanalysis of the IP-MS data revealed GRF4 and GRF7 (table S1), two 14-3-3 protein family members, as putative BZR1-interacting proteins. Notably, 14-3-3 proteins were reported to interact with phosphorylated BZR1 and participate in BZR1 translocation from the nucleus to the cytoplasm (*26*). Because *GRF4* transcript levels were more than 20 times higher than those of *GRF7* in hypocotyls (Fig. 4C), we focused on GRF4 and assessed its interaction with MPKs. We observed an interaction between GRF4 and MPK3/MPK6 by Y2H assay (Fig. 4D), by luciferase complementation imaging (LCI) in *Nicotiana benthamiana* leaves (Fig. 4E), and by BiFC in *Arabidopsis* protoplasts (Fig. 4F). In addition, GRF4 also interacted with the group C MPKs MPK1/MPK2/MPK7 (Fig. 4, D to F), which also play a minor role in auxin-promoted hypocotyl elongation. These results support the hypothesis that MPK3/MPK6 might phosphorylate GRF4 and abolish its imposed regulation on BZR1 subcellular localization.

### GRF4 represses auxin-induced hypocotyl elongation

**Fig. 5.**
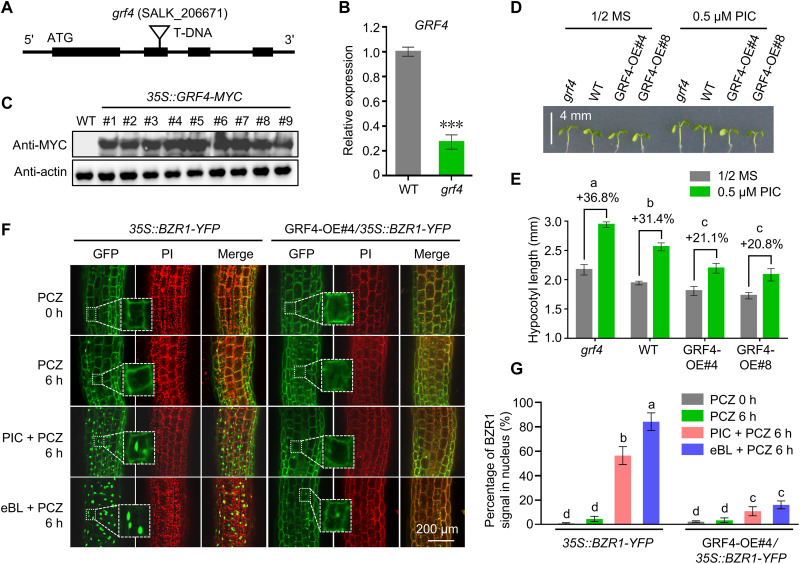
GRF4 negatively regulates hypocotyl elongation. (**A**) Schematic diagram of the *GRF4* locus, with the insertion site of transferred DNA (T-DNA) in the *grf4* mutant. ATG, translation initiation site. (**B**) Relative *GRF4* transcript levels in WT and *grf4* seedlings grown on half-strength MS medium for 6 days. ***P < 0.001 by Student’s *t* test. (**C**) GRF4-MYC protein abundance in different *35S::GRF4-MYC* lines, as determined by immunoblot with an anti-MYC antibody. (**D**) Hypocotyl phenotypes of WT, *grf4*, *35S::GRF4-MYC#4*, and *35S::GRF4-MYC#8* seedlings grown on half-strength MS medium with or without 0.5 μM PIC for 6 days. Scale bar, 4 mm. (**E**) Mean hypocotyl length of the seedlings shown in (D). The percentages indicate the promoting effect of auxin on hypocotyl elongation. *P* < 0.05 by one-way ANOVA. (**F**) Subcellular localization of BZR1-YFP in the hypocotyl cells of *35S::BZR1-YFP* and GRF4-OE#4/*35S::BZR1-YFP* (F1 generation) seedlings grown on half-strength MS medium with 0.2 μM PCZ for 6 days and then treated with 0.2 μM PCZ, 1 μM PIC + 0.2 μM PCZ, or 1 μM eBL + 0.2 μM PCZ for 6 hours. Dotted lines indicate enlarged images. Scale bar, 200 μm. (**G**) Percentage of BZR1-YFP signal in the nucleus based on quantification of the signal in (F) by ImageJ software. *P* < 0.05 by one-way ANOVA.

To uncover the role of GRF4 in auxin-induced hypocotyl elongation, we identified a *grf4* mutant with a transferred DNA (T-DNA) inserted in the second exon (Fig. 5A) with a 75% reduction in *GRF4* transcript levels (Fig. 5B). We also generated lines overexpressing *GRF4* fused to a MYC tag (*GRF4-*OE; Fig. 5C). Unexpectedly, although the 14-3-3 family consists of 13 isoforms (*51*), the *grf4* single mutant exhibited a long hypocotyl phenotype compared to the WT (Fig. 5, D and E), highlighting the central role of GRF4 in 14-3-3-regulated hypocotyl elongation. In contrast to the phenotype of *grf4*, *GRF4-*OE#4 and *GRF4-*OE#8 lines had shorter hypocotyls (Fig. 5, D and E). The promoting effect of PIC on hypocotyl elongation was more pronounced in *grf4* but weaker in *GRF4-*OE#4 and *GRF4-*OE#8 (Fig. 5, D and E), indicating that GRF4 represses auxin-induced hypocotyl elongation.

The small synthetic peptide R18 tightly binds to 14-3-3s and blocks their function (*52*), providing an independent means of inhibiting 14-3-3 activity. Exogenous application of R18 enhanced PIC-induced hypocotyl elongation (fig. S3, A and B), confirming the negative role of 14-3-3 in auxin-triggered hypocotyl elongation. Furthermore, the effect of R18 treatment on hypocotyl elongation was comparable to that seen in the *grf4* single mutant (Fig. 5, D and E), supporting the central role of GRF4 among 14-3-3 family members in regulating hypocotyl growth. A recent report showed that BZR1^S173A^, a variant of BZR1 that cannot interact with 14-3-3, is retained in the nucleus (*26*), demonstrating that 14-3-3 is required for the accumulation of BZR1 in the cytosol. In agreement with this observation, R18 treatment promoted the relocation of cytosolic BZR1 to the nucleus (fig. S3, C and D), concomitantly with enhanced hypocotyl elongation (fig. S3, A and B). More directly, GRF4 accumulation severely impaired PIC- and eBL-induced BZR1 nuclear accumulation (Fig. 5, F and G), reaffirming the unique role of GRF4 in regulating BZR1 subcellular localization. These data support an opposite effect of GRF4 and auxin on hypocotyl elongation, as well as on BZR1 subcellular localization. We conclude that auxin-induced BZR1 nuclear accumulation and hypocotyl elongation are realized by antagonizing the retention of BZR1 imposed by GRF4 to the cytoplasm.

### MPK3/MPK6 phosphorylate GRF4 at residue S248

**Fig. 6.**
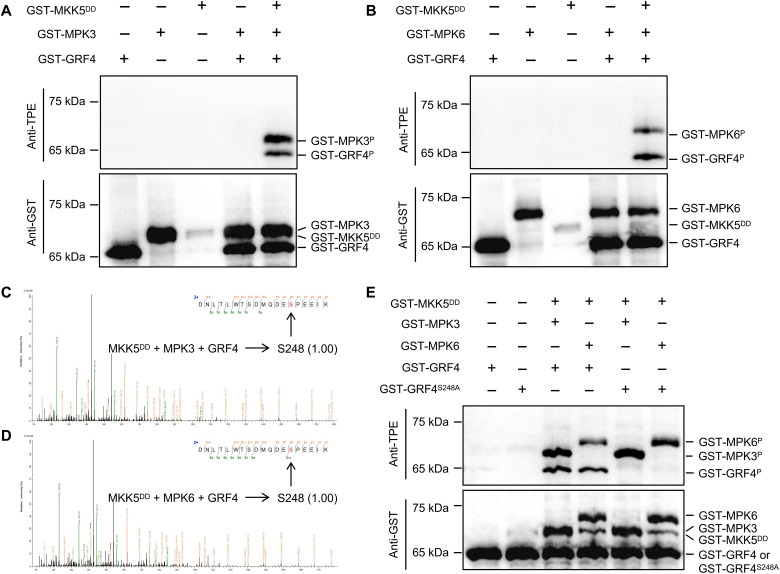
MPK3 and MPK6 share the S248 phosphorylation site of GRF4. (**A** and **B**) In vitro kinase assays showing the phosphorylation of GRF4 by MPK3/MPK6 in the presence or absence of MKK5^DD^. Recombinant GST-MKK5^DD^, GST-MPK3/GST-MPK6, and GST-GRF4 fusion proteins were added into the in vitro reaction buffer in the indicated combinations for 1 hour. The anti–thiophosphate ester–specific antibody (anti-TPE) antibody was used to visualize phosphorylated proteins, with the anti-GST antibody providing a control for protein loading. (**C** and **D**) MS/MS spectra of the phosphorylated GRF4 peptide derived from phosphorylation-mass spectrum showing the phosphorylation site contained in the peptide and the score or confidence of the site. (**E**) In vitro kinase assay showing the phosphorylation of GRF4 but not GRF4^S248A^ by MPK3/MPK6 in the presence or absence of MKK5^DD^. Recombinant GST-MKK5^DD^, GST-MPK3 or GST-MPK6, and GST-GRF4 or GST-GRF4^S248A^ fusion proteins were added into the in vitro reaction buffer in the indicated combinations for 1 hour. The anti-TPE antibody was used to visualize the phosphorylated proteins, with the anti-GST antibody providing a control for protein loading.

Because auxin-activated MPK3/MPK6 interact with GRF4 (Fig. 4, D to F), we hypothesized that MPK3/MPK6 might phosphorylate GRF4 and abolish its inhibition of BZR1 nuclear accumulation. To explore this possibility, we purified recombinant glutathione *S*-transferase (GST)–tagged proteins GST-MKK5^DD^, GST-MPK3, GST-MPK6, and GST-GRF4 in *Escherichia coli* and performed in vitro kinase assays. The anti–thiophosphate ester–specific antibody (anti-TPE), which specifically recognizes phosphorylated proteins (*53*, *54*), revealed the phosphorylation of MPK3 and GRF4 in the presence of MKK5^DD^, while MPK3 alone did not phosphorylate GRF4 (Fig. 6A). We obtained the same results with MPK6 (Fig. 6B), indicating that MKK5-activated MPK3 and MPK6, in turn, phosphorylate GRF4.

To identify the GRF4 site(s) phosphorylated by MPK3 and MPK6, we performed phosphorylation-mass spectrum assays. We detected a unique and consistent phosphorylation site, S248, in both MKK5^DD^-MPK3-GRF4 and MKK5^DD^-MPK6-GRF4 in vitro reactions (Fig. 6, C and D), which conformed to the preferred recognition motif serine-proline of MPKs (*55*). We mutated S248 to the similarly sized and nonphosphorylatable residue alanine in GRF4^S248A^ and repeated the in vitro phosphorylation assays. GRF4^S248A^ was no longer phosphorylated by MPK3 or MPK6 (Fig. 6E), indicating that S248 of GRF4 is the only phosphorylation site recognized by both MPK3 and MPK6. This result also confirmed the redundancy of MPK3 and MPK6.

### Phosphorylation destabilizes GRF4

Phosphorylation has various effects on target proteins, including changes in enzymatic activity, subcellular localization, protein interaction, and protein stability (*56*). Transient transfection of *35S::GFP-GRF4*, *35S::GFP-GRF4^S248A^*, and *35S::GFP-GRF4^S248D^* constructs in *Arabidopsis* protoplasts demonstrated that the phosphorylation state of GRF4 does not affect its subcellular localization (fig. S4A). We independently obtained similar results when transiently infiltrating the same constructs in *N. benthamiana* leaves (fig. S4B). Because 14-3-3 can interact with and regulate the localization of BZR1 (*27*), we asked whether GRF4 phosphorylation status affects its interaction with BZR1, but we determined that BZR1 interacts with GRF4 regardless of phosphorylation (fig. S4C).

**Fig. 7.**
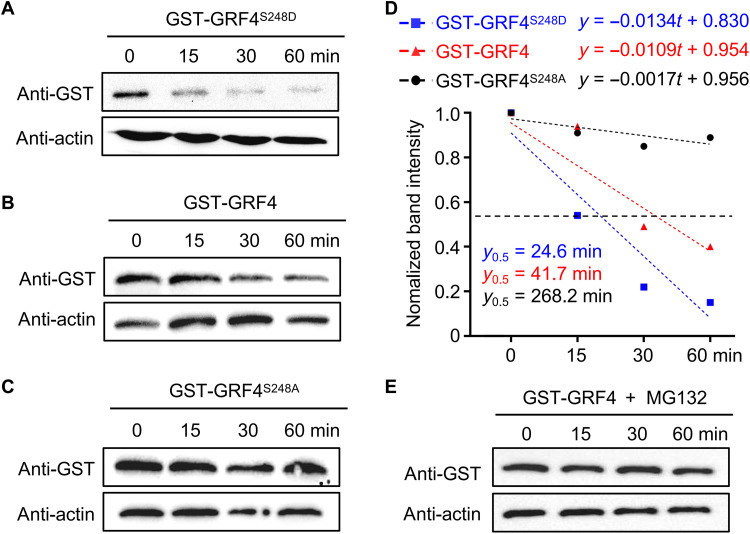
Phosphorylation reduces the protein stability of GRF4. (**A** to **C**) Degradation rates of GST-GRF4^S248D^ (A), GST-GRF4 (B), and GST-GRF4^S248A^ (C) in cell-free assays from WT protein extracts. Recombinant purified GST-GRF4^S248D^, GST-GRF4, and GST-GRF4^S248A^ were added to the protein extracts and incubated for 15, 30, or 60 min. Protein abundance was determined with an anti-GST antibody. (**D**) Linear regressions of the quantified band intensity from (A) to (C) by ImageJ represent the degradation rates of GST-GRF4^S248D^, GST-GRF4, and GST-GRF4^S248A^. (**E**) GST-GRF4 is not degraded in the presence of 200 μM MG132 in cell-free assays. Recombinant GST-GRF4 was incubated with total protein extracts from WT and treated with MG132 for 15, 30, or 60 min. The protein abundance of GST-GRF4 was determined with an anti-GST antibody.

To explore whether phosphorylation might affect GRF4 protein stability, we generated a phosphomimic GRF4 variant, whereby the S248 residue was mutated to aspartic acid (D) in GRF4^S248D^. We then measured the degradation rates of recombinant GST-GRF4^S248A^, GST-GRF4, and GST-GRF4^S248D^ incubated with total protein extracts from WT seedlings in a cell-free system. Recombinant GST-GRF4^S248D^ appeared to be degraded much faster than GST-GRF4 (Fig. 7, A and B), whereas GST-GRF4^S248A^ was more stable (Fig. 7, B and C), suggesting that phosphorylation destabilizes GRF4. We estimated the half-life of each protein variant based on the band intensity of immunoblots: The half-life of the recombinant proteins dropped from 41.7 min with GST-GRF4 to 24.6 min with GST-GRF4^S248D^ (Fig. 7D), while GST-GRF4^S248A^ had an extended half-life of 268.2 min (Fig. 7D). These results indicated that phosphorylation at the S248 residue plays a key role in controlling GRF4 protein stability. We further established that GRF4 is degraded via the 26*S* proteasome, as the addition of the 26*S* proteasome inhibitor MG132 completely blocked GRF4 degradation (Fig. 7E).

### Auxin-induced degradation of GRF4 depends on MPK3/MPK6

**Fig. 8.**
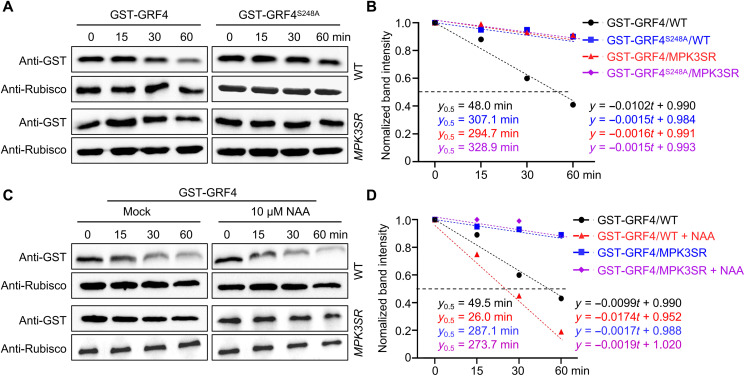
Auxin destabilizes GRF4 via MPK3/MPK6. (**A**) Degradation rates of GST-GRF4 and GST-GRF4^S248A^ in cell-free assays from total protein extracts for WT or *MPK3SR* seedlings. Recombinant purified GST-GRF4 or GST-GRF4^S248A^ was added to WT or *MPK3SR* protein extracts and incubated for 15, 30, or 60 min. Protein abundance was determined with an anti-GST antibody. (**B**) Linear regression of the quantified band intensity from (A) by ImageJ represents the degradation rates of GST-GRF4 and GST-GRF4^S248A^ in WT or *MPK3SR* extracts. *y*_0.5_ represents the time point at which half of the protein is degraded. (**C**) Degradation rates of GST-GRF4 in cell-free assays from total protein extracts for WT or *MPK3SR* in the presence or absence of NAA. Recombinant purified GST-GRF4 was added to WT or *MPK3SR* protein extracts with or without 10 μM NAA and incubated for 15, 30, or 60 min. Protein abundance of GST-GRF4 was determined with an anti-GST antibody. (**D**) Linear regressions of the quantified band intensity from (C) by ImageJ represent the degradation rates of GST-GRF4 in WT or *MPK3SR* in the presence or absence of NAA.

Because MPK3/MPK6-mediated phosphorylation destabilizes GRF4 (Figs. 6 and 7), the degradation of GRF4 should be diminished or absent in the *MPK3SR* line. Recombinant GST-GRF4 was more stable in total protein extracts from the *MPK3SR* line than in WT extracts (Fig. 8A), with a half-life increasing from 48.0 to 294.7 min (Fig. 8B). This observation supported the notion that GRF4 degradation depends on MPK3/MPK6. The degradation of recombinant GST-GRF4^S248A^ was also completely blocked in WT extracts (Fig. 8, A and B), indicating that the loss of MPK3/MPK6 function is equivalent to blocking the phosphorylation of GRF4, both of which substantially increased GRF4 protein stability. These results further confirmed that S248, whose phosphorylation is mediated by MPK3/MPK6, is the key residue that regulates GRF4 stability.

Is MPK3/MPK6-mediated GRF4 degradation triggered by auxin? To answer this question, we repeated the cell-free assays in the presence of NAA, which revealed that NAA markedly accelerates the degradation of recombinant GST-GRF4 in total protein extracts from the WT (Fig. 8C). Specifically, NAA treatment shortened the half-life of GST-GRF4 from 49.5 to 26.0 min (Fig. 8D), almost the same as the 24.6 min seen for GST-GRF4^S248D^ (Fig. 7, A and D). By contrast, NAA-induced GRF4 degradation was blocked in total protein extracts from the *MPK3SR* line (Fig. 8, C and D), indicating that auxin-induced GRF4 degradation is mediated by MPK3/MPK6. With the reduction of GRF4 protein, the interactions between BZR1 and MPK3/MPK6 were notably attenuated (fig. S5).

### GRF4 destabilization is required for auxin-induced hypocotyl elongation

**Fig. 9.**
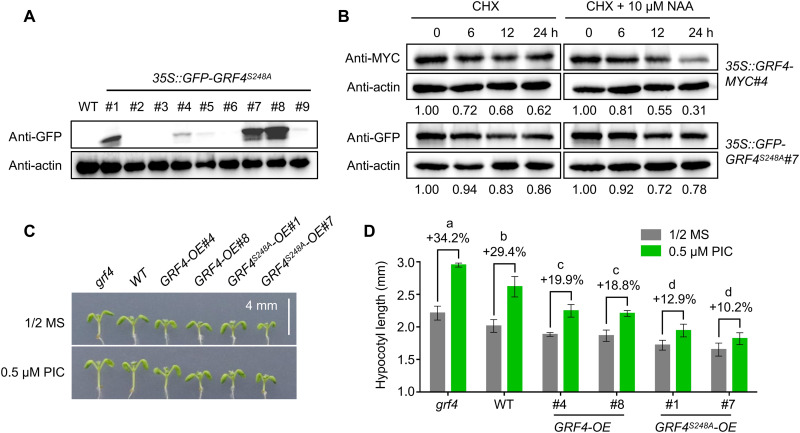
Inability to degrade GRF4 blocks auxin-induced hypocotyl elongation. (**A**) GRF4^S248A^ protein abundance in different *35S::GFP-GRF4^S248A^* lines, as determined by immunoblot with an anti-GFP antibody. (**B**) Comparison of the in vivo degradation rates of GRF4 and GRF4^S248A^ with cycloheximide (CHX) or CHX + NAA treatment. Ten-day-old *35S::GRF4-MYC#4* and *35S::GFP-GRF4^S248A^#7* seedlings were treated with 200 μM CHX or 200 μM CHX + 10 μM NAA for 6, 12, or 24 hours. (**C**) Hypocotyl phenotypes of WT, *grf4*, *GRF4*-OE#4, *GRF4*-OE#8, *GRF4^S248A^*-OE#1, and *GRF4^S248A^*-OE#7 seedlings grown on half-strength MS medium with or without 0.5 μM PIC for 6 days. Scale bar, 4 mm. (**D**) Mean hypocotyl length of the seedlings shown in (C). The percentages indicate the promoting effect of auxin on hypocotyl elongation. *P* < 0.05 by one-way ANOVA.

To assess the contribution of auxin-induced GRF4 degradation to auxin-induced hypocotyl elongation, we generated lines overexpressing *GRF4^S248A^* (Fig. 9A) and compared the degradation rates of GRF4 and GRF4^S248A^ with and without NAA treatment. We first blocked protein translation with cycloheximide (CHX) and then treated seedlings with NAA, which accelerated GRF4 degradation, but not that of the highly stable GRF4^S248A^ (Fig. 9B), indicating that auxin induces the degradation of GRF4 in vivo. To further examine whether auxin-triggered GRF4 destabilization is necessary for auxin-induced hypocotyl elongation, we compared the inhibitory effect of GRF4 and GRF4^S248A^ on PIC-induced hypocotyl elongation: *GRF4* or *GRF4^S248A^* overexpression both inhibited the promoting effect of PIC on hypocotyl elongation, although the effect was more pronounced with *GRF4^S248A^* (Fig. 9, C and D). The excessive stability of GRF4^S248A^ was thus not conducive to hypocotyl elongation, because it strongly represses BZR1 activation of *PRE1* (fig. S6). Therefore, auxin-triggered GRF4 destabilization is essential for auxin-induced hypocotyl elongation.

## DISCUSSION

### BZR1 is a key factor mediating auxin-induced hypocotyl elongation

Auxin-induced hypocotyl elongation was almost lost in the *bzr1* mutant (Fig. 1, A and B), underscoring the essential role of BZR1 in auxin-induced hypocotyl elongation. We also established that combined PIC and PCZ treatments promote the nuclear accumulation of BZR1 (Fig. 1, D and E), suggesting that auxin deploys BZR1 to regulate hypocotyl elongation even in the presence of low BR signaling in vivo. Although the auxin and BR signaling pathways may cross-talk at multiple nodes, BZR1 is undoubtedly a critical mediator of auxin-induced hypocotyl elongation. Because BZR1-mediated BR signaling regulates hypocotyl elongation by interacting with ARF6, and ARF6 shares almost half of its target genes with BZR1 (*57*), it will be interesting to investigate whether auxin can affect the formation of the ARF6-BZR1 dimer in the future.

### MPK-mediated auxin signaling in hypocotyl elongation

TMK1/TMK4-mediated phosphorylation of AHA2 provides insights into auxin-induced hypocotyl elongation (*22*). The MKK4/MKK5-MPK3/MPK6 module acts downstream of TMK1/TMK4 to regulate lateral root formation (*40*), but whether this module also acts downstream of TMK1/TMK4 for hypocotyl elongation remains unclear. Our results show that the *MKK5^DD^*-OE line exhibits enhanced auxin-induced hypocotyl elongation, while *mpk3-1*, *mpk6-3*, *MPK3SR*, and *mpk6^−/−^mpk3RNAi-est* displayed shorter hypocotyls and reduced auxin-induced hypocotyl elongation (Fig. 3, D to I). Therefore, we hypothesize that the TMK1/4-MKK4/5-MPK3/6 signaling pathway is also active in auxin-induced hypocotyl elongation. In addition, the *mpk1 mpk2 mpk14* triple mutant also attenuated auxin-induced hypocotyl elongation (Fig. 3, B and C), suggesting that at least group A and group C MPKs are involved in auxin-induced hypocotyl elongation.

### BIN2 and MPKs differentially regulate BZR1 subcellular localization

Phosphorylation of BZR1 by BIN2 is a prerequisite signal to regulate the subcellular localization of BZR1. However, without 14-3-3, both phosphorylated and nonphosphorylated BZR1 localize to the nucleus (*26*), highlighting the important role of 14-3-3 proteins in regulating BZR1 subcellular localization. BR treatment rapidly promotes BZR1 nuclear accumulation by decreasing BIN2-mediated phosphorylation of BZR1. In this study, we provide evidence that auxin-activated MPKs promote BZR1 nuclear accumulation via the phosphorylation and destabilization of 14-3-3 (Figs. 2 and 6 to 9). The 14-3-3 family consists of 13 members (*51*), but the knockdown *grf4* mutant described in this study already exhibited a clear hypocotyl phenotype (Fig. 5), suggesting that GRF4 plays a major role in hypocotyl elongation. This result also provides evidence that different 14-3-3 isoforms recognize different target proteins and thus participate in different biological processes (*58*, *59*), with certain cell types having very specific requirements for specific 14-3-3 isoforms (*51*).

**Fig. 10.**
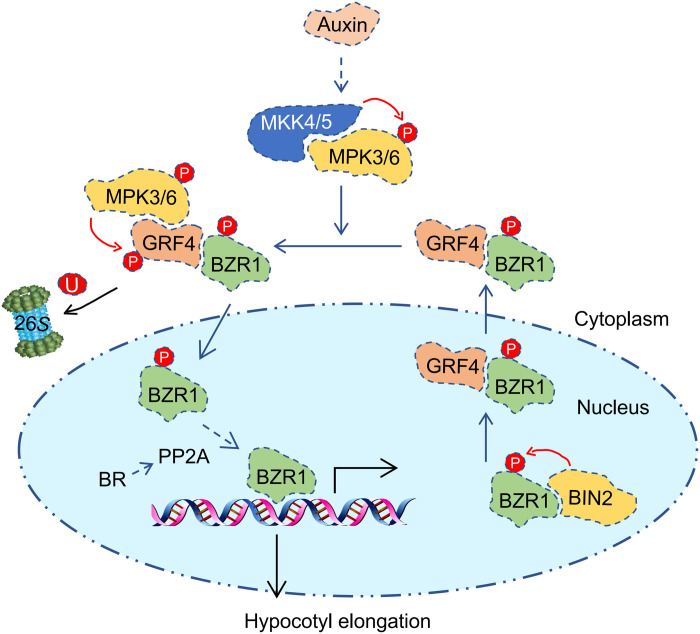
Auxin promotes BZR1 nuclear accumulation to induce hypocotyl elongation. After phosphorylation by BIN2, phosphorylated-BZR1 (BZR1^P^) is captured by 14-3-3 and transported from the nucleus to the cytoplasm where it remains. Auxin-activated MPK3/MPK6 phosphorylate GRF4 at residue S248 and trigger the degradation of GRF4 by the 26*S* proteasome. The released BZR1^P^ enters the nucleus and is converted to BZR1 by BR-activated protein phosphatase 2A (PP2A), allowing it to regain transcriptional activity and induce the expression of genes involved in cell elongation and hypocotyl elongation.

Another clue worth mentioning is that the promotion effect of auxin on hypocotyl elongation is notably weakened in *bri1-116* mutant (*4*), suggesting that the activation of BZR1 by auxin requires the assistance of BR in some necessary link. We hypothesized that the dephosphorylation of BZR1 after entering the nucleus was still dependent on BR-activated protein phosphatase 2A (PP2A) (*28*). Together, we propose a possible working model to explain how auxin promotes BZR1 nuclear accumulation during hypocotyl elongation (Fig. 10): BZR1, phosphorylated by BIN2, is transported from the nucleus to the cytoplasm by 14-3-3 proteins and is retained in the cytoplasm, while the auxin-activated MKK5-MPK3/MPK6 module phosphorylates GRF4 and guides its degradation through the 26*S* proteasome. Released BZR1 reenters the nucleus and is dephosphorylated by BR-activated PP2A, which allows it to regain transcriptional activity and induce hypocotyl elongation.

### Auxin regulates BZR1 subcellular localization in a tissue-dependent manner

As has long been known, auxin can promote or inhibit growth in a concentration- and organ-dependent manner (*60*); however, the underlying mechanism has not been revealed. In this study, we provide evidence that, in contrast to auxin-inhibited BZR1 nuclear accumulation in the root stem cell niche (*31*), auxin enhances BZR1 nuclear localization in hypocotyl cells (Fig. 1, D and E), suggesting that the effects of auxin on BZR1 subcellular localization are tissue or organ specific. There may be a tissue- or cell-specific regulator, which is absent in hypocotyl cells, that can be activated by auxin and confine BZR1 to the cytoplasm in the root stem cell niche. Alternatively, auxin may enhance BZR1 nuclear accumulation in hypocotyl cells via hypocotyl cell–specific factors that are absent in roots. In addition, the different endogenous auxin concentrations between the root stem cell niche and the hypocotyl might also account for this opposite effect of auxin on BZR1 subcellular localization.

Besides regulating BZR1 nuclear accumulation, auxin appears to slightly enhance the protein stability of BZR1 (fig. S1B), presumably due to auxin-mediated 14-3-3 degradation. 14-3-3 can interact with phosphorylated BZR1 (*27*) and thus attenuate its protein stability (*61*), suggesting that 14-3-3 can only promote the degradation of phosphorylated BZR1. Consistent with this, NAA mainly enhanced the protein stability of phosphorylated BZR1 (fig. S1B), indicating that auxin enhanced the protein stability of BZR1 mainly through degradation of 14-3-3. Nonetheless, although NAA can enhance the protein stability of BZR1, this effect is obviously weak, compared with the promotion of its accumulation in the nucleus. In summary, this study reveals the molecular mechanism by which auxin promotes hypocotyl elongation by enhancing BZR1 nuclear accumulation via MPK3/MPK6-regulated GRF4 protein stability.

## MATERIALS AND METHODS

### Plant materials

All plant materials used in this study are in the Columbia-0 background. The mutants and overexpression materials include *mpk3-1* (*44*), *mpk6-3* (*45*), *MPK3SR* (*mpk3 mpk6 MPK3pro:MPK3^TG^*) (*47*), *mpk6^−/−^mpk3RNAi-est* (*40*), *MKK5^DD^-OE* (*48*), *mpk9* (SALK_064439) and *mpk1 mpk2 mpk14* (*38*), and *35S::BZR1-YFP* and *bzr1* (*34*). The *grf4* T-DNA insertion mutant (SALK_206671) was obtained from the AraShare mutant library (www.arashare.cn/index/Product/index.html); the genotyping primers are listed in table S2. The coding sequence of *GRF4* was cloned into the pSuper1300-MYC vector to construct *35S::GRF4-MYC*, and the coding sequence of *GRF4^S248A^* was cloned into the pB7WGF2.0 vector to obtain *35S::GFP-GRF^S248A^*. The S248A mutation was introduced in the *GRF4* coding sequence by polymerase chain reaction (PCR), using the primers listed in table S2.

### Hypocotyl elongation analysis

To determine the hypocotyl phenotype of different plant materials in the presence or absence of auxin, all materials were grown on half-strength Murashige and Skoog (MS) medium with or without 0.5 μM the auxin analog PIC (Aladdin, Shanghai, China) for 6 days under low-light conditions at 22°C with a 16-hour light/8-hour dark photoperiod. To test the effect of MPKs on auxin-induced hypocotyl elongation, 5 μM U0126 (Sigma-Aldrich, Saint Louis, MO, USA) or EGF (50 ng/ml; Sigma-Aldrich) was added to half-strength MS medium with or without 0.5 μM PIC to inhibit or activate MKK activity, respectively. To obtain the *mpk3 mpk6* double mutants, 0.5 μM NA-PP1 (Toronto Research Chemicals, Toronto, Ontario, Canada) was added to the culture medium for *MPK3SR* and 0.02 μM EST (Sigma-Aldrich) was added to the growth medium for *mpk6^−/−^mpk3RNAi-est*. To overexpress *MKK5^DD^*, *MKK5^DD^*-OE seedlings were grown on half-strength MS medium containing 0.02 μM dexamethasone (DEX) (Sigma-Aldrich) or 0.02 μM DEX + 0.5 μM PIC for 6 days. To detect the effect of 14-3-3 on auxin-induced hypocotyl elongation, the R18 peptide was synthesized according to the previously reported sequence PHCVPRDLSWLDLEANMCLP (*52*) by Beijing Dingguo Changsheng Biotechnology Co. Ltd. (Beijing, China) and added at a concentration of 1 or 10 μM to half-strength MS medium with or without 0.5 μM PIC. Hypocotyl lengths of all genotypes were measured with ImageJ software (version 1.46r).

### Subcellular localization analysis of BZR1

To determine the effect of auxin on BZR1 localization, *35S::BZR1-YFP* seedlings were grown on half-strength MS medium containing 0.2 μM PCZ (an inhibitor of BR biosynthesis; Sigma-Aldrich) for 6 days and then transferred to half-strength liquid MS medium containing 0.2 μM PCZ + 1 μM PIC or 0.2 μM PCZ + 1 μM eBL (Sigma-Aldrich) for 3, 6, or 12 hours. The localization of BZR1-YFP in hypocotyl cells was observed with a confocal microscope (Zeiss LSM 880, Germany). To investigate the effect of MPKs on the localization of BZR1, 6-day-old *35S::BZR1-YFP* seedlings were transferred to half-strength MS liquid medium containing 0.2 μM PCZ, 0.2 μM PCZ + 1 μM PIC, 0.2 μM PCZ + 1 μM PIC +10 μM U0126, or 0.2 μM PCZ + 1 μM PIC + EGF (100 ng/ml) for 6 hours. Similarly, to investigate the effect of 14-3-3 on the localization of BZR1, 6-day-old *35S::BZR1-YFP* seedlings were transferred to half-strength MS liquid medium containing 0.2 μM PCZ, 0.2 μM PCZ + 1 μM PIC, or 0.2 μM PCZ + 1 μM PIC +10 μM R18 for 6 hours. The signal intensity of BZR1-YFP in the nucleus and the signal intensity of the entire cell were quantified by ImageJ to calculate the percentage of BZR1 signal in the nucleus.

### RNA extraction and RT-qPCR

To detect the expression levels of various genes in hypocotyls, about 50 mg of hypocotyls was collected from WT seedlings grown for 6 days in half-strength MS medium with or without 0.5 μM PIC. Total RNA was extracted using an RNeasy Plant Mini Kit (QIAGEN, Hilden, Germany). After determining the RNA concentration, 1 μg of RNA was reverse-transcribed into first-strand complementary DNA (cDNA) according to the Transcriptor First Strand cDNA Synthesis Kit instructions (Roche, Basel, Switzerland). Quantitative PCR (qPCR) was performed on a MyiQ Real-time PCR Detection System (Bio-Rad, Hercules, CA, USA) using the ChamQ SYBR Color qPCR Master Mix (Q411, Vazyme, Nanjing, China). *ACTIN2* and *UBQ10* were used as the reference controls. All primers used for reverse transcription (RT)–qPCR are listed in table S2.

### BiFC assays

For BiFC assay, the coding sequence of *GRF4* was cloned in-frame with the sequence of *cEYFP* in the pSAT6-cEYFP-C1 vector to construct *cEYFP-GRF4*, and the coding sequences of *MPK*s were cloned in-frame with the sequence of *nEYFP* in the pSAT6-nEYFP-C1 vector to construct *nEYFP-MPK*s. The plasmid combinations *nEYFP-MPK*s + *cEYFP-GRF4*, *nEYFP* + *cEYFP-GRF4*, and *nEYFP-MPK*s + *cEYFP* were transfected into *Arabidopsis mesophyll* protoplasts, cultured at 25°C for 12 hours in the dark, and then transferred to normal light/dark conditions for 48 hours. For protoplasts preparation, refer to the details previously described (*62*). In brief, 20 mM MES lysis buffer [400 mM mannitol, 20 mM KCl, 1.5% (w/v) Cellulase RS, and 0.4% (w/v) Y-23] was prepared and treated in a 55°C water bath for 10 min. After being cooled to room temperature, pH with KOH was adjusted to 5.7, 10 mM CaCl2 and 0.1% bovine serum albumin were added, and the solution was filtered with a 0.45-μm sieve for backup. Leaves of 4-week-old, nonflowering Arabidopsis were cropped and collected. The lower epidermis was removed with adhesive tape and placed in the MES lysis buffer, which was hydrolysated in darkness for 2 to 3 hours until the protoplasts dissociated from the leaves. An equal volume of W5 solution (2 mM MES, 5 mM KCl, 154 mM NaCl, and 125 mM CaCl_2_) was added to stop the reaction, and the solution was filtered through a 75-μm sieve into a 50-ml centrifuge tube. The supernatant was removed by centrifugation at 100*g* for 2 min at 4°C. W5 solution (10 ml) was added, lightly mixed, and left on ice for 30 min. At 4°C, centrifugation was performed at 100*g* for 2 min to remove the supernatant, and then, MMG (4 mM MES, 15 mM MgCl_2_, and 400 mM mannitol) was added to resuspend the protoplasts. About 2 μg of different combinations of plasmids were added to 100 μl of protoplasts, and 110 μl of polyethylene glycol (PEG) solution (200 mM mannitol and 100 mM CaCl_2_) was added after gently mixing. The reaction was performed in darkness for 15 min, and then, 440 μl of W5 solution was added and left for 5 min. Centrifugation was performed at 100*g* at 4°C for 2 min to remove supernatant. Then, 1 ml of WI solution (4 mM MES, 400 mM mannitol, and 15 mM MgCl_2_) was added and transferred to a 12-well plate for dark culture for 16 hours. A confocal microscope (Zeiss LSM 880, Germany) was used to observe fluorescence. Primers used for BiFC are listed in table S2.

### LCI assays

For LCI assay, the coding sequence of *GRF4* was cloned in-frame with the sequence of *nLUC* in the JW771 vector to construct *GRF4-nLUC*, and the coding sequences of *MPK*s were cloned in-frame with the *cLUC* sequence in the JW772 vector to construct *cLUC-MPK*s. The plasmids and empty vectors were individually transformed into *Agrobacterium* (*Agrobacterium tumefaciens*) strain GV3101 and infiltratred into four different regions of the 30-day-old *N. benthamiana* leaves in the following pairs: *GRF4-nLUC* + *cLUC-MPK*s, *nLUC* + *cLUC-MPK*s, *GRF4-nLUC* + *cLUC*, and *nLUC* + *cLUC*. Attention was paid to avoid overlapping infiltrated leaf areas. After growth in darkness for 12 hours, the infiltrated *N. benthamiana* plants were transferred to light and grown at 26°C for 48 hours. The leaves were sprayed with d-luciferin potassium salt (Meilunbio, Dalian, China) for 5 min, and the luminescence was observed with a Tanon-5200 Multi imaging system (Tanon, Shanghai, China). Primers used for cloning are listed in table S2.

### Y2H assays

For Y2H assays, the coding sequences of the relevant target genes were cloned into pGBKT7 and pGADT7 vectors and transformed into Y2H yeast strain according to the PEG/lithium acetate method (Takara, Japan). Positive transformants were selected on synthetic defined (SD) medium without leucine and tryptophan and grown at 30°C for 2 days. The positive clones were then spotted onto SD medium lacking leucine, tryptophan, adenine, and histidine for 2 to 3 days to test the protein-protein interaction. Primers used for cloning of Y2H constructs are listed in table S2.

### Protein IP, LC-MS/MS, and data analysis

For protein IP, 10-day-old *35S::BZR1-YFP* and *35S::GFP* seedlings were ground into powder with liquid nitrogen; 4 g of powder was immediately transferred to a precooled 50-ml centrifuge tube and resuspended in 4 ml of precooled lysis buffer [1% (v/v) Triton X-100, 150 mM NaCl, 50 mM tris-HCl (pH 7.5), 5 mM EDTA, and a protease inhibitor cocktail tablet]. The samples were incubated at 4°C for 40 min, followed by centrifugation at 14,000*g* for 15 min at 4°C. The supernatant was then transferred to a new 50-ml centrifuge tube, to which 50 μl of agarose beads coupled with anti–green fluorescent protein (GFP) antibody (Smart-Lifesciences, Changzhou, China) was added and slowly rotated at 4°C for 4 hours. The beads were collected by centrifugation at 500*g* for 3 min at 4°C, and the beads were washed with lysis buffer three times.

For liquid chromatography–tandem MS (LC-MS/MS), the IP beads were sent to Shanghai Bioprofile (Shanghai, China) for analysis. The bound proteins were extracted from beads using SDT lysis buffer [4% (w/v) SDS, 100 mM dithiothreitol (DTT), and 100 mM tris-HCl (pH 8.0)]. In brief, the beads were boiled for 3 min, followed by sonication and centrifugation at 16,000*g* for 15 min to remove undissolved beads. The collected supernatant containing proteins was digested with 2 μg of trypsin (Promega, Madison, WI, USA) at 37°C for 12 hours, followed by centrifugation at 16,000*g* for 15 min to collect peptides, and then desalinated with C18 StageTip. LC-MS/MS analysis was performed on a Q Exactive HF-X mass spectrometer coupled to Easy nLC1200 (Thermo Fisher Scientific, Waltham, MA, USA). The raw data were searched against the Uniprot Protein Database (136,783 total entries, downloaded 11 April 2021) using MaxQuant 1.6.1.0 software. The database search results were filtered and exported with <1% false discovery rate (FDR) at the peptide-spectrum–matched level and protein level. The MaxQuant software was set with parameters: for MS spectra accuracy of 20 parts per million (ppm; first round of search) and 4.5 ppm (second round of search), MS/MS spectra accuracy of 0.5 Da, and with options “match between runs” and “re-quantify” enabled.

### In vitro kinase assay

The coding sequences of *MKK5^T215D/S221D^* (*MKK5^DD^*) (*48*), *MPK3*, *MPK6*, *GRF4*, and *GRF4^S248A^* were cloned into the pGEX-4T-1 vector to add a GST tag. The plasmids were transformed into *E. coli* BL21, and the production of recombinant GST-MKK5^DD^, GST-MPK3, GST-MPK6, GST-GRF4, and GST-GRF4^S248A^ was induced with 0.5 mM isopropyl β-d-1-thiogalactopyranoside at 16°C for 12 hours and then purified with Glutathione Sepharose 4 Fast Flow (GE Healthcare, Chicago, IL, USA). The in vitro kinase assay was performed as described previously (*49*). In brief, 0.1 μg of recombinant GST-MKK5^DD^, 0.2 μg of GST-MPK3 or GST-MPK6, and 2 μg of GST-GRF4 or GST-GRF4^S248A^ were incubated in a 30-μl kinase reaction [10 mM MgCl_2_, 1 mM adenosine 5′-*O*-(3-thiotriphosphate), 50 mM tris-HCl (pH 7.5), and 1 mM DTT] at 23°C for 30 min. Then, 20 mM EDTA and 1.5 mM *p*-nitrobenzyl mesylate (ABclonal, Wuhan, China) were added and incubated at 23°C for another 30 min, and 5× loading buffer was used to terminate the reaction. Anti-TPE (ABclonal, Wuhan, China), an antibody that specifically recognizes phosphorylated proteins (*53*, *54*), was used to determine whether MPK3 and MPK6 can phosphorylate GRF4 and GRF4^S248A^, and the anti-GST antibody (TransGen, Beijing, China) was used to determine whether the loading quantity of each protein was consistent.

### Phos-MS identification of phosphorylation residues of GRF4

To identify the GRF4 residues phosphorylated by MPK3 and MPK6, an in vitro kinase assay was conducted by incubating recombinant GST-MKK5^DD^ + GST-MPK3 + GST-GRF4 and GST-MKK5^DD^ + GST-MPK6 + GST-GRF4, as above. After terminating the reaction, the samples were sent to Shanghai Bioprofile (Shanghai, China) for phos-MS analysis. The enzymatic hydrolysis, desalting, and sequencing of phos-MS were as described for LC-MS/MS analysis above, with phosphorylation added as a peptide modification. The scores and reliability of each site were obtained by analyzing the abundance of peptide segments and frequency of phosphorylated sites. For MPK3/MPK6, this study only focused on serine and threonine residues as phosphorylated sites. For identification, peptides of at least seven amino acids were required. An FDR of 1% for peptide and protein identification was independently applied. Similar protein sequences that did not permit distinction were clustered into groups of proteins.

### Cell-free assay and protein half-life calculation

Cell-free experiments were performed as previously described (*63*, *64*). In brief, 14-day-old WT and *MPK3SR* seedlings were ground into powder in liquid nitrogen, followed by transfer of 1.2 g of powder to a precooled 5.0-ml centrifuge tube and the addition of 1.2 ml of nondenaturating protein extraction buffer [10 mM NaCl, 25 mM tris-HCl (pH 7.5), 4 mM phenylmethylsulfonyl fluoride, 10 mM adenosine 5′-triphosphate, 5 mM DTT, and 10 mM MgCl_2_] and incubation at 4°C for 40 min. After centrifugation at 4°C at 17,000*g* for 10 min, 1.2 ml of supernatant was collected and divided into three 2.0-ml centrifuge tubes. Then, 16 μl of recombinant GST-GRF4^S248A^, GST-GRF4, or GST-GRF4^S248D^ was added to one of the three centrifuge tubes and incubated at 4°C for 0, 15, or 30 min or 1 hour. At each time point, 100 μl of the reaction solution was taken out, and 25 μl of 5× loading buffer was added to stop the reaction. Immunoblotting was used to detect the abundance of GST-GRF4^S248A^, GST-GRF4, and GST-GRF4^S248D^ at different time points in WT and *MPK3SR* samples. Signal intensity was quantified by ImageJ software, with the abundance at 0 min set to 1. The half-life of the protein refers to the time when half of the protein is degraded (*63*, *64*). From the results obtained with ImageJ, the slope “*k*” and constant “*b*” of the linear regression equation were calculated with Statistical Product and Service Solutions (SPSS, version 24) software, to obtain the formula *y* = *kt* + *b*, where “*t*” represents time and “*y*_0.5_” represents the time corresponding to half protein degradation.

### Statistical analysis

Statistical analyses were performed using Student’s *t* test (*P < 0.05; **P < 0.01; ***P < 0.001) or one-way analysis of variance (ANOVA; *P* < 0.05; least significant difference and Duncan test) in SPSS software (version 24). All experiments had at least three biological replicates, and the data were presented as means ± SE.

## Notes

View/request a protocol for this paper from *Bio-protocol*.
