# Supplementary material for: Auxin promotes hypocotyl elongation by enhancing BZR1 nuclear accumulation in Arabidopsis
Source: Sci Adv. 2023 Jan 4;9(1):eade2493. doi: 10.1126/sciadv.ade2493 (PMC9812374; doi:10.1126/sciadv.ade2493)
Supplement: Supplementary file 1 — Figs. S1 to S6 Tables S1 and S2 [file sciadv.ade2493_sm.pdf]

Science Advances
AAAS

Supplementary Materials for

Auxin promotes hypocotyl elongation by enhancing BZR1 nuclear accumulation in *Arabidopsis*

Zipeng Yu *et al.*

Corresponding author: Zhaojun Ding, dingzhaojun@sdu.edu.cn

Sci. Adv. **9**, eade2493 (2023)
DOI: 10.1126/sciadv.ade2493

This PDF file includes:

Figs. S1 to S6
Tables S1 and S2

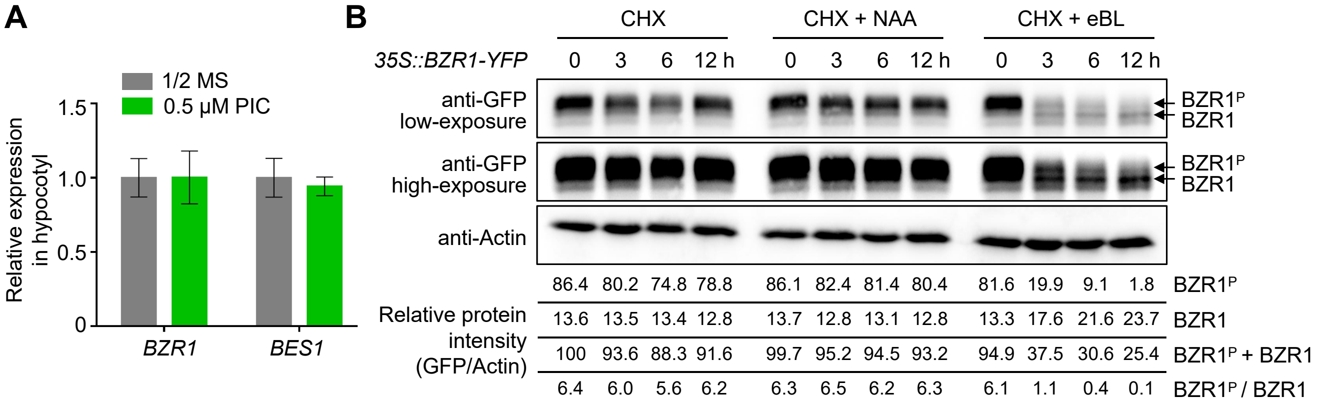

Supplementary Figure 1. Effects of auxin on BZR1 transcription, protein stability, and phosphorylation status

(A) Relative *BZR1* and *BES1* transcript levels in hypocotyls of the WT grown on half-strength MS medium with or without 0.5 μM PIC for 6 days. **(B)** BZR1 protein abundance and phosphorylation proportion in 6-day-old *35S::BZR1-YFP* seedlings treated with 200 μM cycloheximide (CHX), 200 μM CHX + 10 μM 1-naphthaleneacetic acid (NAA), or 200 μM CHX + 1 μM eBL for 3, 6, or 12 h. The protein intensity was quantified by ImageJ software.

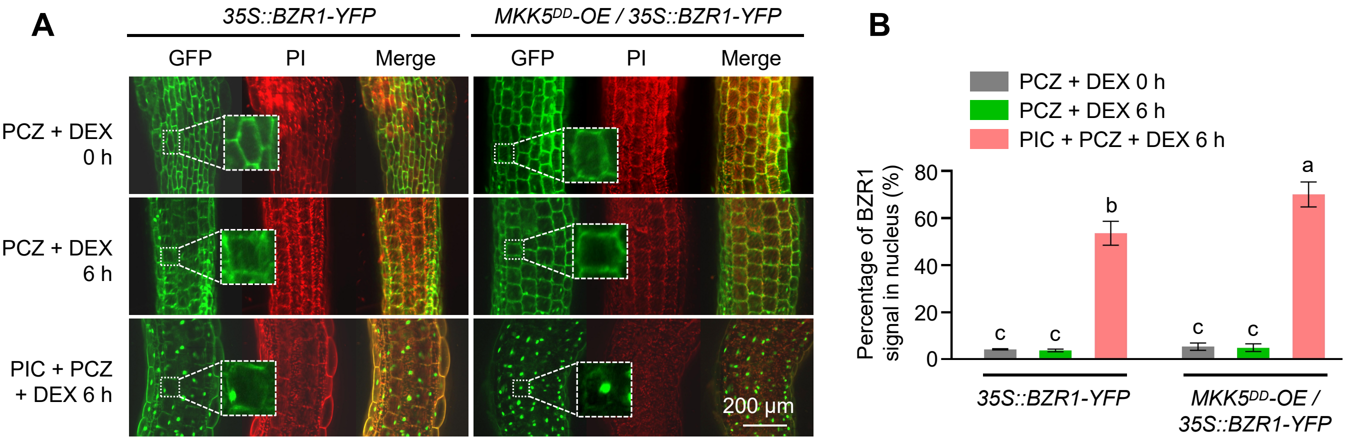

Supplementary Figure 2. MKK5^{DD} enhances auxin-induced BZR1 nuclear accumulation

(A) Subcellular localization of BZR1-YFP in the hypocotyl cells of *35S::BZR1-YFP* and *MKK5^{DD}-OE* / *35S::BZR1-YFP* (F1 generation) seedlings grown on half-strength MS medium with 0.2 μM PCZ + 0.02 μM DEX for 6 days and treated with 0.2 μM PCZ + 0.2 μM DEX or 1 μM PIC + 0.2 μM PCZ + 0.2 μM DEX for 6 h. Dotted lines indicate enlarged images. Scale bar = 200 μm. **(B)** Percentage of BZR1-YFP signal in the nucleus based on quantification of the signal in (A) by ImageJ software. $P<0.05$; by one-way ANOVA.

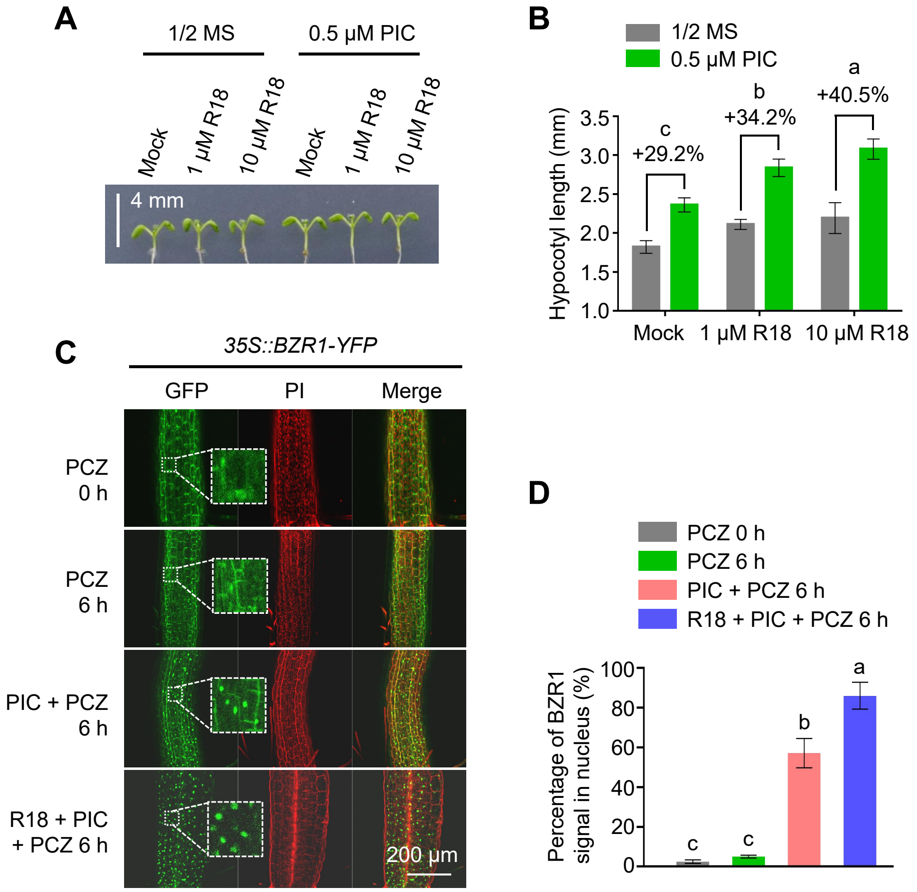

Supplementary Figure 3. 14-3-3s repress auxin-induced hypocotyl elongation and BZR1 nuclear accumulation

(A) Hypocotyl phenotypes of WT seedlings treated with various concentrations of R18 peptide and grown on half-strength MS medium with or without 0.5 μM PIC for 6 days. Scale bar = 4 mm. **(B)** Mean hypocotyl length of the seedlings shown in (A). The percentages indicate the promoting effect of auxin on hypocotyl elongation. P<0.05; by one-way ANOVA. **(C)** Subcellular localization of BZR1-YFP in the hypocotyl cells of *35S::BZR1-YFP* seedlings grown on half-strength MS medium with 0.2 μM PCZ for 6 days and then treated with 0.2 μM PCZ, 1 μM PIC + 0.2 μM PCZ, or 10 μM R18 + 1 μM PIC + 0.2 μM PCZ for 6 h. Dotted lines indicate enlarged images. Scale bar = 200 μm. **(D)** Percentage of BZR1-YFP signal in the nucleus based on quantification of the signal in (C) by ImageJ software. P<0.05; by one-way ANOVA.

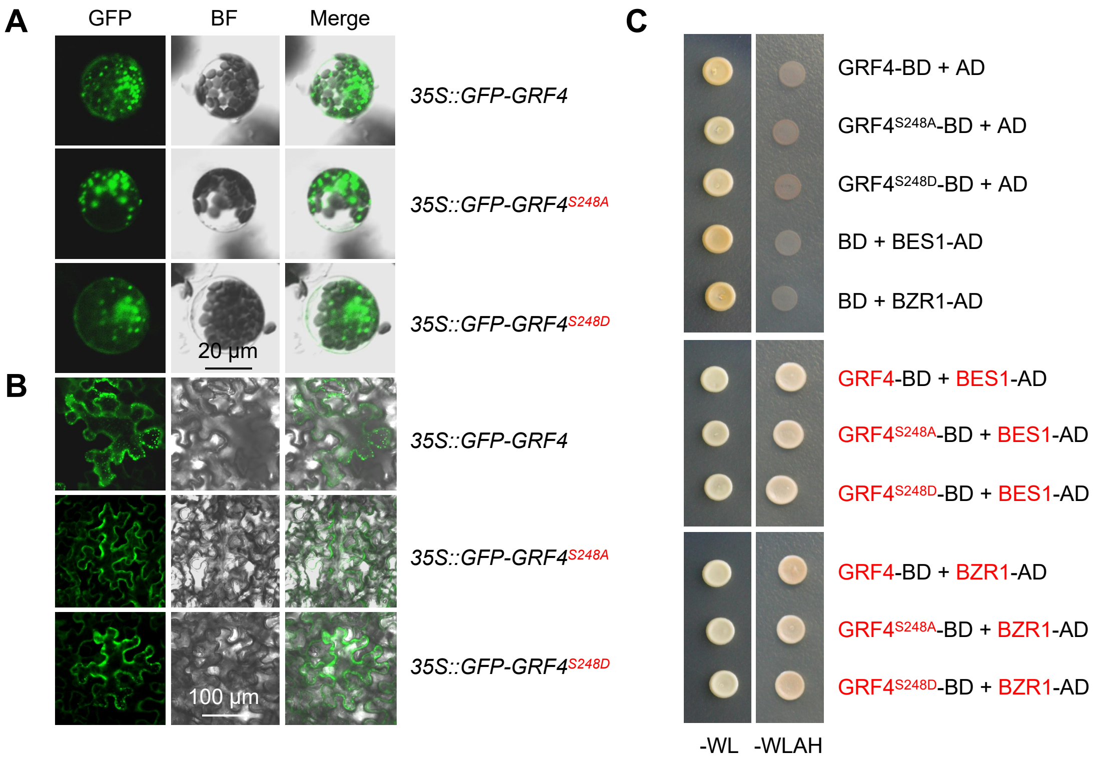

Supplementary Figure 4. Effects of phosphorylation on GRF4 localization and interaction with BZR1

(A) Transient transformation experiment performed in Arabidopsis protoplasts shows subcellular localization of GFP-GRF4, GFP-GRF4^{S248A}, and GFP-GRF4^{S248D}. Scale bar = 20 μm. **(B)** Transient transformation experiment performed in *Nicotiana benthamiana* leaves shows subcellular localization of GFP-GRF4, GFP-GRF4^{S248A}, and GFP-GRF4^{S248D}. Scale bar = 100 μm. **(C)** Y2H validation of BZR1 and BES1 (also known as BZR2, a homologous protein of BZR1) interaction with GRF4, GRF4^{S248A}, and GRF4^{S248D}, respectively.

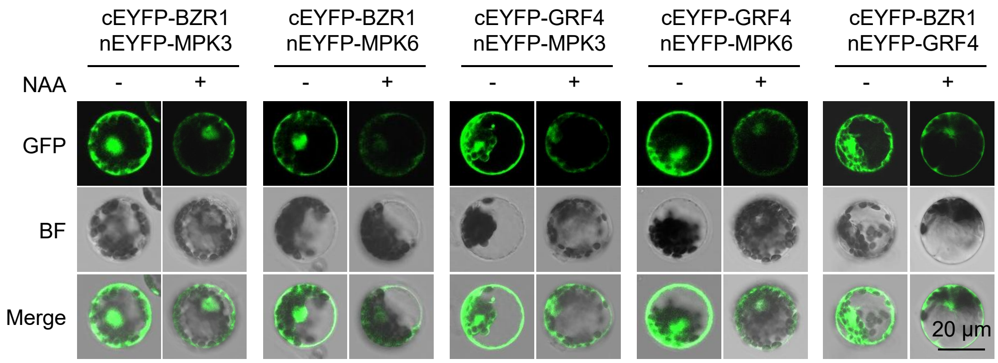

Supplementary Figure 5. Auxin reduces pairwise interactions between BZR1, GRF4, and MPK3/MPK6

BiFC assay performed in transfected Arabidopsis protoplasts showing the interactions between MPK3/MPK6, GRF4, and BZR1. The effect of auxin on the interaction strength of these proteins was tested by treating protoplasts with or without 2 μM NAA for 12 h. Scale bar = 20 μm.

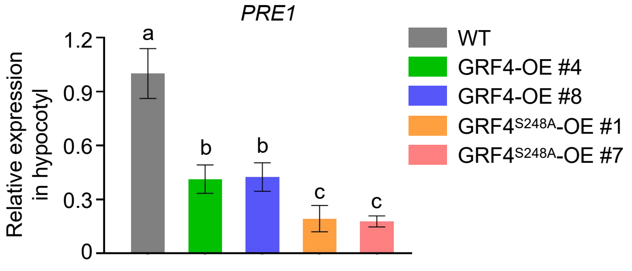

Supplementary Figure 6. GRF4 represses *PRE1* expression in hypocotyl cells

Relative *PRE1* transcript levels in hypocotyls of the WT, GRF4-OE #4, GRF4-OE #8, $GRF4^{S248A}$-OE #1, and $GRF4^{S248A}$-OE #7 grown on half-strength MS medium for 6 days. P<0.05; by one-way ANOVA.

Supplementary Table 1. Candidate BZR1-interacting proteins identified in IP-MS

Protein	Relative intensity 35S::BZR1-YFP Repeat-1	Relative intensity 35S::BZR1-YFP Repeat-2	Intensity 35S::GFP Repeat-1	Intensity 35S::GFP Repeat-2
GFP	833773146	1470229515	1048300000	1255700000
BZR1	492693203	462157667	0	0
BZR2/BES1	204346380	205510589	0	0
MPK1	3017962	2098791	0	0
GRF7; 14-3-3-like protein GF14 nu	2788336	3430203	0	0
GF14 PHI; GRF4; 14-3-3-like protein GF14 phi	2543297	3729571	0	0
TPR1; TPR2; TPR3; TPR4	1526999	1297483	0	0
ATMPK3, MPK3	1243708	2554607	0	0
CYP74A	812607	559075	0	0
DOX1	693447	1329748	0	0
At2g24270;ALDH11A3	479843	509779	0	0
RPS10	261393	213704	0	0
ACO3	218916	230757	0	0
CAD5	213782	266559	0	0
SBP1;SBP2	209025	116232	0	0
UBP12;UBP13	204601	98251	0	0
CML12;At2g41100	189988	240282	0	0
BIN2/ASK7	175807	137088	0	0
CLPP2	169897	170462	0	0
RABE1C;RABE1D;At5g59840;RABD1;RABD2C; RABD2A;RABE1A;RABD2B;RABE1E	169753	143264	0	0
GAMMA-ADR	157423	22763	0	0
HSP70-9	154507	49127	0	0
NOP5-2	139395	109761	0	0
RPS2B;RPS2A;RPS2C;RPS2D	136601	48497	0	0
NHO1;GLPK;At1g80460	120967	176350	0	0
XI-K;At5g20470	110051	144506	0	0
At5g42765	96487	147600	0	0
At4g17100	95886	67957	0	0
ACS	80695	125657	0	0
sc35; SC35	73832	100931	0	0
THFS	70434	80082	0	0
JAO2	61620	52883	0	0
At3g59020	59336	80050	0	0
ALY3	57725	40694	0	0
SSI2;FAB2	55995	123916	0	0
At4g16060	51415	39800	0	0
KAB1	51220	89243	0	0
XPO7	49124	57735	0	0
VPS18	38112	26582	0	0
At5g48790	28750	41788	0	0
VIP1	21941	92485	0	0

Supplementary Table 2. Primers used in this study

Purpose	Name	Sequence (5'-3')
grf4 mutant identification	LBb3.1	GTTTGGCTTTGAATTTCTCTG
	GRF4-LP	CCTAAAAACCGACCCAAAAAG
	GRF4-RP	TTCGTTTCGATCGTTTACCTG
Mutation of S248 residue of GRF4 protein	BD-GRF4-F	GGAATTCCATATGATGGCGGCACCACCAGCATCAT
	BD-GRF4-R	CGGGATCCGATCTCCTTCTGTTCTTCAGCAGGC
	BD-GRF4^{S248A}-R	CGGGATCCGATCTCCTTCTGTTCTTCAGCAGGCTTTGGTGCTGCTGCTTCTTTAATCTCCTCCGGAGCT
	BD-GRF4^{S248D}-R	CGGGATCCGATCTCCTTCTGTTCTTCAGCAGGCTTTGGTGCTGCTGCTTCTTTAATCTCCTCCGGATCT
GST-GRF4 expressed in *Escherichia coli*	GRF4-pGEX4t-1-F	CCGCGTGGATCCCCGATGGCGGCACCACCAGCATCAT
	GRF4-pGEX4t-1-R	ATGCGGCCGCTCGAGGATCTCCTTCTGTTCTTCAGCAGGC
qRT-PCR	qPCR-GRF4-F	GTTTGGCTTTGAATTTCTCTG
	qPCR-GRF4-R	ACTATCCTTGTATGACTCCTC
	qPCR-GRF7-F	TCTTAACTTCTCTGTCTTCTACT
	qPCR-GRF7-R	ACTGTCTTTGTATGATTCTTCTC
	qPCR-BZR1-F	CATTCCTTCTTCTCTTCCTTC
	qPCR-BZR1-R	TTAGCGATAGATTCCCAGTT
	qPCR-BES1-F	TGAAGAAGACGGAACTACTT
	qPCR-BES1-R	CTGACTTGGTAAGATAAGATGG
	qPCR-BEE1-F	GCTTTGTTACCTACTTTGTCT
	qPCR-BEE1-R	TCGTCTTCTTCTTCCTTCTT
	qPCR-PRE1-F	AATGATTGACCTCGTATCTAAG
	qPCR-PRE1-R	GTATGTAATTGCATGTCTCTTG
	qPCR-MPK1-F	CTGGTAACCTTCTTGTCAAC
	qPCR-MPK1-R	GAGTCACAACATATTCAGTCAT
	qPCR-MPK2-F	TTCAGCAAACATTCTCCATC
	qPCR-MPK2-R	CTAGTCACAACATATTCAGTCAT
	qPCR-MPK3-F	CTTGCTAGACCTACTTCAGA
	qPCR-MPK3-R	ATACAACCAACAGACCAAAC
	qPCR-MPK4-F	CTGCTACTTAATTGCTCTGAA
	qPCR-MPK4-R	GATGAGTCTAAGCTGATGAAC
	qPCR-MPK5-F	CAAGGACTACATCAGAAACAG
	qPCR-MPK5-R	CAACCAACAGACCATACATC
	qPCR-MPK6-F	CCAAGTAATCTCCTCCTGAA
	qPCR-MPK6-R	TACCATCTCGTGACAACATA
	qPCR-MPK7-F	GATGTTATGTTGCCTGCTAA
	qPCR-MPK7-R	AGAAAGAGACTGAGAGGATTT
	qPCR-MPK8-F	CAACACAGCCAATTTCAAAG
	qPCR-MPK8-R	AAGGTATTCCTCCAACATCT
	qPCR-MPK9-F	CAGTTCCATTCACTCACAAA
	qPCR-MPK9-R	TTGCCAGACCATAGAAGTAA
	qPCR-MPK10-F	ACTTGCTCATCCATACCTTA
	qPCR-MPK10-R	GGCTTCACAGTAGATTAACTC
	qPCR-MPK11-F	GCGATCAAGAAGATTGGTAAT
	qPCR-MPK11-R	TGAGGTGGTCTTATTATGTCTAT
	qPCR-MPK12-F	TGAAATTAAACTTCTCAGGCATA
	qPCR-MPK12-R	TCAGTGTCCATTAACTCATAGA
	qPCR-MPK13-F	AGCAACTTGGTTCTGAATAC
	qPCR-MPK13-R	CATCTCGTCACAACATACTC
continued...	continued...	continued...

Purpose	Name	Sequence (5'-3')
qRT-PCR	qPCR-MPK14-F	CTTGCGAGAACTTATGAACA
	qPCR-MPK14-R	AAGAATCTCTGCGAATATGC
	ACTIN2-F	GAGAGATTCAGATGCCCAGAAG
	ACTIN2-R	GGTGCAAGTGCTGTGATTTC
	UBQ10-F	GCCAAGATCCAGGACAAGG
	UBQ10-R	CGCAGGACCAAGTGAAGAG
BiFC	GRF4-CEYFP-F	CGAGCTCAAGCTTCCATGGCGGCACCACCAG
	GRF4-CEYFP-R	GACTCTAGATCAGGTGATCTCCTTCTGTTCTTCAGCAGG
	MPK5-NEYFP-F	CGAGCTCAAGCTTCCATGGCGAAGGAAATTGAATCAGC
	MPK5-NEYFP-R	GACTCTAGATCAGGTAATGCTCGGCAGAGGATTGAACT
	MPK7-NEYFP-F	CGAGCTCAAGCTTCCATGGCGATGTTAGTTGAGCC
	MPK7-NEYFP-R	GACTCTAGATCAGGTGGCATTTGAGATTTCAGCT
	MPK9-NEYFP-F	CGAGCTCAAGCTTCCATGGATCCTCATAAAAAGGTTG
	MPK9-NEYFP-R	GACTCTAGATCAGGTAGTGTGGAGAGCCGCGA
	MPK1-NEYFP-F	CGAGCTCAAGCTTCCATGGCGACTTTGGTTGATCC
	MPK1-NEYFP-R	GACTCTAGATCAGGTGAGCTCAGTGTTTAAGGT
	MPK2-NEYFP-F	CGAGCTCAAGCTTCCATGGCGACTCCTGTTGATCC
	MPK2-NEYFP-R	GACTCTAGATCAGGTAAACTCAGAGACCTCATTG
	MPK3-NEYFP-F	CGAGCTCAAGCTTCCATGAACACCGGCGGTGGC
	MPK3-NEYFP-R	GACTCTAGATCAGGTCTAACCGTATGTTGGATTGAGTGC
	MPK6-NEYFP-F	CGAGCTCAAGCTTCCATGGACGGTGGTTCAGGTCA
	MPK6-NEYFP-R	GACTCTAGATCAGGTCTATTGCTGATATTCTGGATTGAAA
	MPK4-NEYFP-F	CGAGCTCAAGCTTCCATGTCGGCGGAGAGTTGTTTCGG
	MPK4-NEYFP-R	GACTCTAGATCAGGTCACTGAGTCTTGAGGATTGAACTTGACTGT
	GRF4-NEYFP-F	CGAGCTCAAGCTTCCATGGCGGCACCACCAGCATC
	GRF4-NEYFP-R	GACTCTAGATCAGGTGATCTCCTTCTGTTCTTCAGCAGGCT
LCI	GRF4-771-F	cgggggacgagctcggtaccATGGCGGCACCACCAG
	GRF4-771-R	acgagatctggtcgacGATCTCCTTCTGTTCTTCAGCAGGC
	MPK1-772-F	acgcgtcccggggcggtaccATGGCGACTTTGGTTGATCC
	MPK1-772-R	agctctgcaggtcgacTCAGAGCTCAGTGTTTAAGGT
	MPK2-772-F	acgcgtcccggggcggtaccATGGCGACTCCTGTTGATCC
	MPK2-772-R	agctctgcaggtcgacTCAAAACTCAGAGACCTCATTG
	MPK3-772-F	acgcgtcccggggcggtaccATGAACACCGGCGGTGGC
	MPK3-772-R	agctctgcaggtcgacCTAACCGTATGTTGGATTGAGTGCT
	MPK4-772-F	acgcgtcccggggcggtaccATGTCGGCGGAGAGTTGTTTCGG
	MPK4-772-R	agctctgcaggtcgacCACTGAGTCTTGAGGATTGAACTTGACTGT
	MPK5-772-F	acgcgtcccggggcggtaccATGGCGAAGGAAATTGAATCAGCG
	MPK5-772-R	agctctgcaggtcgacTTAAATGCTCGGCAGAGGATTGAACT
	MPK6-772-F	acgcgtcccggggcggtaccATGGACGGTGGTTCAGGTCA
	MPK6-772-R	agctctgcaggtcgacCTATTGCTGATATTCTGGATTGAAAGCAA
	MPK7-772-F	acgcgtcccggggcggtaccATGGCGATGTTAGTTGAGCC
	MPK7-772-R	agctctgcaggtcgacTTAGGCATTTGAGATTTCAGCT
	MPK9-772-F	acgcgtcccggggcggtaccATGGATCCTCATAAAAAGGTTG
	MPK9-772-R	agctctgcaggtcgacTCAAGTGTGGAGAGCCGCGA
continued...	continued...	continued...

Purpose	Name	Sequence (5'-3')
Y2H	BD-MPK3-F	ATGGCCATGGAGGCCATGAACACCGGCGGTGGC
	BD-MPK3-R	CCGCTGCAGGTCGACCTAACCGTATGTTGGATTGAGTGC
	BD-MPK6-F	ATGGCCATGGAGGCCATGGACGGTGGTTCAGGTCA
	BD-MPK6-R	CCGCTGCAGGTCGACCTATTGCTGATATTCTGGATTGAA
	BD-GRF4-F	ATGGCCATGGAGGCCATGGCGGCACCACCAGCATCAT
	BD-GRF4-R	CCGCTGCAGGTCGACGATCTCCTTCTGTTCTTCAGCAGG
	AD-MPK1-F	GCCATGGAGGCCAGTATGGCGACTTTGGTTGATCC
	AD-MPK1-R	CAGCTCGAGCTCGATTCAGAGCTCAGTGTTTAAGGT
	AD-MPK2-F	GCCATGGAGGCCAGTATGGCGACTCCTGTTGATCC
	AD-MPK2-R	CAGCTCGAGCTCGATTCAAAACTCAGAGACCTCATTG
	AD-MPK3-F	GCCATGGAGGCCAGTATGAACACCGGCGGTGGC
	AD-MPK3-R	CAGCTCGAGCTCGATCTAACCGTATGTTGGATTGAGTGC
	AD-MPK4-F	GCCATGGAGGCCAGTATGTCGGCGGAGAGTTGTTTCGG
	AD-MPK4-R	CAGCTCGAGCTCGATCACTGAGTCTTGAGGATTGAA
	AD-MPK5-F	GCCATGGAGGCCAGTATGGCGAAGGAAATTGAATC
	AD-MPK5-R	CAGCTCGAGCTCGATAATGCTCGGCAGAGGATTGAAC
	AD-MPK6-F	GCCATGGAGGCCAGTATGGACGGTGGTTCAGGTCA
	AD-MPK6-R	CAGCTCGAGCTCGATCTATTGCTGATATTCTGGATTG
	AD-MPK7-F	GCCATGGAGGCCAGTATGGCGATGTTAGTTGAGCC
	AD-MPK7-R	CAGCTCGAGCTCGATTTAGGCATTTGAGATTTCAGCT
	AD-MPK8-F	GCCATGGAGGCCAGTATGGGTGGTGGTGGGAATCT
	AD-MPK8-R	CAGCTCGAGCTCGATAGAATTGTGAAGAGAAGCAA
	AD-MPK9-F	GCCATGGAGGCCAGTATGGATCCTCATAAAAAGGTTG
	AD-MPK9-R	CAGCTCGAGCTCGATTCAAGTGTGGAGAGCCGCGA
	AD-MPK10-F	GCCATGGAGGCCAGTATGGAGCCAACTAACGATGC
	AD-MPK10-R	CAGCTCGAGCTCGATATCATTGCTGGTTTCAGGGT
	AD-MPK11-F	GCCATGGAGGCCAGTATGTCAATAGAGAAACCATT
	AD-MPK11-R	CAGCTCGAGCTCGATAGGGTTAAACTTGACTGATT
	AD-MPK12-F	GCCATGGAGGCCAGTATGTCTGGAGAATCAAGCTCT
	AD-MPK12-R	CAGCTCGAGCTCGATGTGGTCAGGATTGAATTTGAC
	AD-MPK13-F	GCCATGGAGGCCAGTATGGAGAAAAGGGAAGATGG
	AD-MPK13-R	CAGCTCGAGCTCGATCATATTCTTGAAGTGTAAAG
	AD-MPK14-F	GCCATGGAGGCCAGTATGGCGATGCTAGTTGATCC
	AD-MPK14-R	CAGCTCGAGCTCGATAGCTCGGGGGAGGTAATGAA
	AD-BZR1-F	GCCATGGAGGCCAGTATGACTTCGGATGGAGCTACG
	AD-BZR1-R	CAGCTCGAGCTCGATACCACGAGCCTTCCCATTTCC
	AD-BES1-F	GCCATGGAGGCCAGTATGAAAAGATTCTTCTATAATTCC
	AD-BES1-R	CAGCTCGAGCTCGATACTATGAGCTTTACCATTTCC
Protoplast transformation	35S::BZR1-MYC-F	CAAATCGACTCTAGAATGACTTCGGATGGAGCTACGTCG
	35S::BZR1-MYC-R	actagtatttaaatgACCACGAGCCTTCCCATTTCCA
